# Gender and sex in eating disorders: A narrative review of the current state of knowledge, research gaps, and recommendations

**DOI:** 10.1002/brb3.2871

**Published:** 2023-02-24

**Authors:** Édith Breton, Robert‐Paul Juster, Linda Booij

**Affiliations:** ^1^ CHU Sainte‐Justine Research Centre Montreal Canada; ^2^ Department of Psychiatry and Addictology University of Montreal Montreal Canada; ^3^ Research Centre of the Montreal Mental Health University Institute Montreal Canada; ^4^ Department of Psychology Concordia University Montreal Canada; ^5^ Department of Psychiatry McGill University Montreal Canada

**Keywords:** biological psychiatry, brain development, eating disorders, gender/sex, sexual and gender diversities

## Abstract

**Introduction:**

Eating disorders (EDs) have long been considered conditions exclusively affecting women, and studies in the ED field regularly exclude men. Research efforts are needed to better understand the role of gender and sex in EDs. This review describes the role of gender and sex in the development of EDs from a biopsychosocial perspective.

**Methods:**

The primary hypothesis of this narrative review is that gender and sex interact to influence ED risk. The literature review was conducted using the PubMed database.

**Results:**

This review first presents the general characteristics and prevalence of EDs according to gender and sex. Next, neurodevelopmental processes, neurobiology, gender roles, body image, and the minority stress model are addressed. Lastly, research perspectives to better include gender and sex in the field of EDs are discussed (e.g., representation of gender and sex diversities, development of appropriate assessment tools, and increasing awareness).

**Conclusion:**

Although substantial knowledge gaps remain, there is a growing recognition of the importance of integrating gender and sex in ED research that holds promise for further development in the field.

## Graphical abstract

In this review, we describe the role of sex and gender in the development of eating disorders. An innovative aspect of the present review is that we integrate neurodevelopmental and neurobiological literature with the more well‐document psychosocial theories on the role of gender and sex. We conclude the review with recommendations, in order to provide insights that may improve the integration of sex and gender in eating‐disorder research.

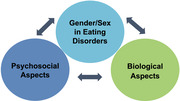

## 1 INTRODUCTION

Sex and gender are closely related constructs that play an essential yet understudied role in the development of *eating disorders* (EDs). Sex refers to biological characteristics, including genetics (e.g., sex chromosomes), physiology (e.g., sex hormones), and anatomy (e.g., the reproductive system) (Clayton & Tannenbaum, 2016; Tannenbaum et al., 2016). Generally, sex is assigned at birth and considered a dichotomous variable; however, intersex individuals are a group of people that transcend sex as a binary construct due to their diversity in sexual characteristics. In contrast, gender refers to a psychosocial phenomenon (Clayton & Tannenbaum, 2016; Swaab et al., 2021; Tannenbaum et al., 2016) that is modulated by environmental forces and can fluctuate throughout life (Tannenbaum et al., 2016). Gender includes gender identity (i.e., how individuals define themselves), gender roles (i.e., social norms and stereotypes related to masculinity and femininity), and gender relations (i.e., interactions between individuals according to their gender and the expectations placed on them by society) (Clayton & Tannenbaum, 2016; Tannenbaum et al., 2016). For further definitions, a glossary of key terms used in this review can be found in Table 1. Given the close interaction between biology and environment, gender and sex are two interacting constructs that must be appropriately considered in research (i.e., they should not be used interchangeably) (Clayton & Tannenbaum, 2016; DuBois et al., 2021; Howard et al., 2017; Tannenbaum et al., 2016). The term gender/sex has been used in previous literature to represent the interaction between gender and sex as well as the recognition that these biological and sociocultural constructs are often intertwined (Hyde et al., 2019).

**TABLE 1:** Glossary of key terms related to sex and gender research^a,b^

**Sex**	Biological attributes of humans and animals that include physical features, chromosomes, gene expression, hormones, and anatomy
**Sex assigned at birth**	Categorization as male or female on one's birth certificate that is based primarily on medical examination of genitals
**Gender**	Socioculturally constructed roles, behaviors, expressions, and identities of girls, women, boys, men, and gender‐diverse people
**Gender/sex**	Term used to recognize that biological sex and sociocultural gender are typically inseparable
**Gender identity**	A person's innate sense of being male, female, both, neither, or even dimensionally
**Gender roles**	Sociocultural stereotypes an individual assimilates, enacts, and adopts throughout life
**Gender relations**	Interactions between people and institutions according to sex and gender identity, based on the expectations placed on them by society
**Cisgender**	Individuals whose gender aligns with their sex assigned at birth.
**Transgender**	Umbrella term that refers to individuals whose gender identity does not align with sex assigned at birth. Some examples include transmasculine, transfeminine, gender nonbinary, genderqueer, gender‐fluid, First Nations two‐spirit individuals, and bigender
**Intersex**	Individuals with variations or combinations of what are considered XY male‐typical and XX female‐typical chromosomal, gonadal, and genital characteristics that do not fit neatly into one binary sex category

^a^Chase, B., & Ressler, P. (2009). An LBGT/queer glossary. The English Journal, 98(4):23−24.

^b^Hyde et al. (2019).

To our knowledge, few studies have considered *both* gender and sex in ED research. EDs have long been considered a condition that primarily affects girls and women (Coelho et al., 2021; Murray et al., 2017; Strother et al., 2012). For example, in *anorexia nervosa* (AN), amenorrhea—a symptom that can only be observed in individuals with a female reproductive system—had been one of the diagnostic criteria up to the publication of the DSM‐IV (American Psychiatric Association, 2013). Even today, woman‐centered conceptualizations of EDs may negatively affect some individuals based on their sex (Hartman‐Munick et al., 2021; Murray et al., 2017; Romito et al., 2021; Strother et al., 2012; Uniacke et al., 2021). Consequently, more efforts are needed to address the role of gender/sex in ED research. Relying on birth‐assigned sex as a binary variable is not sufficient to capture the high levels of inter‐ and intra‐individual variability within the same sex, or to refine the psychosocial aspects of gender diversity that modulate ED risk.

This narrative review describes the role of gender/sex in the development of EDs from a biopsychosocial perspective. Our overarching hypothesis is that both gender and sex, and their *interaction*—as implied by the term gender/sex—influence risk for EDs (which is linked to high levels of symptoms, without the individual necessarily meeting all diagnostic criteria). To address this, evidence based on clinical and community samples were included. The rationale to include research from community samples in addition to research from clinical samples was based on that subclinical levels of ED symptoms are common in the general population (e.g., body dissatisfaction and binge eating behaviors) (Zeiler et al., 2016). Moreover, increased levels of these symptoms are thought to increase the risk of developing full‐threshold ED in some individuals (Stice et al., 2017; Treasure et al., 2015). Thus, subclinical symptoms as studied in community samples were included to highlight potential gender and sex differences in these predisposing aspects of EDs.

**FIGURE 1:**
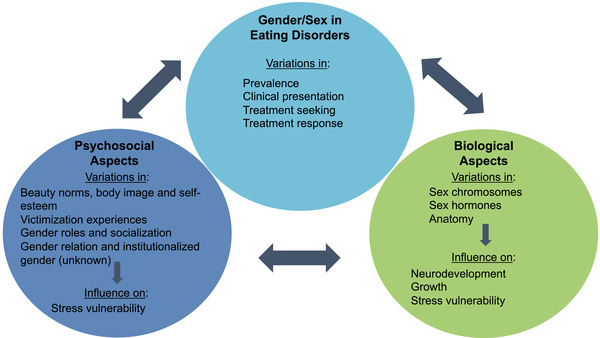
A biopsychosocial perspective on the role of gender/sex in eating disorders

Our review takes a transdiagnostic approach. By doing so, we focus on etiological similarities and differences between different types of EDs (Smolak & Levine, 2015). Although this review focuses mainly on results obtained in human samples, some evidence from animal models has been included, as these paradigms may shed light on certain processes that remain difficult to study in humans (e.g., the effect of hormones on the brain). Figure 1 summarizes the main concepts addressed in the present review. First, the prevalence and characteristics related to gender/sex in EDs will be presented. Next, aspects linked to neurodevelopment, neurobiology, gender roles, body image, and the minority stress model will be addressed. Finally, research perspectives on gender/sex to move EDs research forward will be discussed.

## 2 PREVALENCE AND CHARACTERISTICS OF EATING DISORDERS ACCORDING TO SEX AND GENDER

In the general population, the prevalence of EDs ranges between 1% and 5%, and men represent between 17% and 43% of the cases seen in ED clinics (American Psychiatric Association, 2013; Murray et al., 2017). Hence, the prevalence of EDs is often considered higher in women than in men. Beyond sex, those whose gender identity does not align with their sex assigned at birth (e.g., transgender, genderqueer, gender‐fluid, and gender nonbinary) report a greater prevalence of ED symptoms compared to cisgender individuals (Grammer et al., 2021). For example, the prevalence of EDs among transgender individuals ranges between 2% and 18% (Hartman‐Munick et al., 2021). These differences in prevalence rates suggest that specific risk factors (e.g., minority stress, sociocultural norms, and expectations) may differentially affect risk for disordered eating behaviors according to gender and sex (Bankoff & Pantalone, 2014; Dotan et al., 2021), which further underscores the importance of addressing gender diversity in ED research.

In addition, sexual orientation—a construct closely related to gender and sex, yet orthogonal—is associated with risk for EDs (Bankoff & Pantalone, 2014; Calzo et al., 2018; Dotan et al., 2021; Grammer et al., 2021; Murray et al., 2017; Nagata et al., 2020). Findings from a recent study using data from the Avon Longitudinal Study of Parents and Children (ALSPAC) indicated that gay and bisexual boys had 2.67 times the odds to engage in dieting in order to lose weight at age 14, and 12.53 times the odds of binge eating at age 16 compared to completely heterosexual boys (Calzo et al., 2018). Gay and bisexual boys also reported more body dissatisfaction than their heterosexual peers (Calzo et al., 2018). Moreover, lesbian and bisexual girls had more than three times the odds of binge eating behaviors at ages 14 and 16, and of purging behaviors at age 16 compared to completely heterosexual girls (Calzo et al., 2018). Although there appears to be a consensus that disordered eating is more prevalent in gay and bisexual boys and men than their heterosexual counterparts, findings are more conflicting for lesbian or bisexual girls and women (Bankoff & Pantalone, 2014). These findings reinforce the need for research efforts to better understand EDs in these populations.

Furthermore, the clinical presentation of EDs differs according to gender/sex diversity (Nagata et al., 2020; Roberts et al., 2021). For example, body image concerns in men often relate to muscularity (Coelho et al., 2021; Lavender et al., 2017; Murray et al., 2017; Ridout et al., 2021). Overtraining is also more prevalent in men than in women (Coelho et al., 2021; Lavender et al., 2017; Murray et al., 2017; Ridout et al., 2021). Men are more frequently diagnosed with other specified or unspecified EDs than women (Coelho et al., 2021; Lavender et al., 2017; Murray et al., 2017; Ridout et al., 2021). Men may also be less likely to seek professional help for an ED, and when they do, symptoms are often more severe and comorbidities more frequent relative to women (Murray et al., 2017; Ridout et al., 2021). Beyond the typical differences observed between men and women, subgroup differences might also exist between individuals with different sexual orientations (e.g., lesbian vs. bisexual women).

Relatedly, current diagnostic tools may be more sensitive in detecting EDs in women (Coelho et al., 2021; Franceschini & Fattore, 2021; Hartman‐Munick et al., 2021; Lavender et al., 2017; Murray et al., 2017; Ridout et al., 2021). Indeed, these tools are often based on a clinical presentation of EDs that is perhaps more typical in women (Coelho et al., 2021; Franceschini & Fattore, 2021; Hartman‐Munick et al., 2021; Lavender et al., 2017; Murray et al., 2017; Ridout et al., 2021). This may complicate the detection of EDs in men (Lavender et al., 2017; Strother et al., 2012) and may contribute to the impression that the differences in prevalence between sexes are greater than it really is. There is therefore a need for research efforts to optimize diagnostic tools for EDs, to consider the existing variability related to gender/sex.

## 3 NEURODEVELOPMENT, GENDER, AND SEX

Neurodevelopment and growth are both associated with risk for ED symptoms (e.g., Herle et al., 2020; Marion et al., 2020; Watson et al., 2019). Importantly, these two processes do not occur at the same rate for boys and girls (Culbert et al., 2021; Fisher et al., 2018; Franceschini & Fattore, 2021; McCarthy et al., 2017). Differential rates in neurodevelopment may influence the timing of greater vulnerability to adversity according to sex (Culbert et al., 2021; Franceschini & Fattore, 2021; McCarthy et al., 2017; VanRyzin et al., 2020). In this regard, the endocrine system is an important area of research for understanding sex differences in the risk of EDs, as sex hormones play a major role in critical periods for neurodevelopment—including periods when signs and symptoms of EDs often develop (e.g., puberty) (Culbert et al., 2021; McCarthy et al., 2017; Mikhail et al., 2021; VanRyzin et al., 2020). The role of sex hormones during the prenatal and perinatal periods is also of interest, as sex hormones are involved in the organization of the brain during development, which may promote behavioral, cognitive, and emotional differences between boys and men as well as girls and women (Culbert et al., 2021; McCarthy et al., 2017; Mikhail et al., 2021; VanRyzin et al., 2020). For example, vulnerability to adversity is likely influenced by developmental peaks in sex hormones (e.g., estrogen and testosterone), which may contribute to differentiate boys and girls as it relates to ED (Culbert et al., 2021; Fisher et al., 2018; Franceschini & Fattore, 2021; Hildebrandt et al., 2010; McCarthy et al., 2017; VanRyzin et al., 2020). Studies in animal models suggest that elevated prenatal and perinatal testosterone levels in males compared to females may be protective from binge eating behaviors later in life (Mikhail et al., 2021). Furthermore, studies in animals and in humans suggest that estrogen has an inhibitory effect on food intake (Franceschini & Fattore, 2021; Hildebrandt et al., 2010), and low levels of estrogens have been associated with symptoms of AN and BN (Hildebrandt et al., 2010; Ramoz et al., 2013). The role of estrogen pathways in EDs is also supported by findings from genetic studies, including evidence of polymorphisms in genes relevant to the estrogen pathways (e.g., *ESRRA*, *HDAC4*, and *ESR2*) (Cui et al., 2013; Franceschini & Fattore, 2021; Paolacci et al., 2020; Scott‐Van Zeeland et al., 2014).

The interaction between estrogens and neurotransmission systems (e.g., serotonin system) might play a role in gender/sex differences in EDs (Hildebrandt et al., 2010). Indeed, sex hormones can modulate specific brain pathways implicated in EDs, including those involved in appetite regulation and reward response (Culbert et al., 2021; Franceschini & Fattore, 2021; Lenroot & Giedd, 2010; Swaab & Bao, 2020). This modulation can be sex‐dependent and affect behavior (Culbert et al., 2021; Franceschini & Fattore, 2021; Lenroot & Giedd, 2010; Swaab & Bao, 2020). For example, women typically show a greater preference for high‐calorie and fatty food than men (Culbert et al., 2021). This differential preference is also observed in animal models and is associated with hormonal activity (Culbert et al., 2021; Franceschini & Fattore, 2021; Lenroot & Giedd, 2010). Eating behaviors and nutritional status (e.g., malnutrition) can, in turn, affect the endocrine system, which further complexifies research on the role of sex hormones in EDs (Franceschini & Fattore, 2021; Schorr & Miller, 2017). Moreover, the menstrual cycles (which are associated with variations in hormonal levels) have also been associated with levels of binge eating symptoms (Franceschini & Fattore, 2021; Mikhail et al., 2021). Longitudinal studies are needed to clarify the link between neurodevelopment, hormones, and ED symptoms, especially in boys and girls. Given that interactions between biological processes and environment are closely linked to neuroendocrine functioning, future studies should investigate the link between gender/sex, hormones, and EDs in gender‐diverse populations.

Additionally, an important developmental period of vulnerability to EDs is puberty (Klump, 2013), when the first signs of EDs often tend to appear (Swanson et al., 2011; Treasure et al., 2020). Physical and behavioral differences between boys and girls become more pronounced during puberty (Allen et al., 2008; Favaro et al., 2009; Smink et al., 2013; Stice et al., 2013). The influence of puberty on the risk for EDs is likely due to biological aspects (e.g., hormone levels and rapid body changes), psychosocial aspects (e.g., the stress of entering high school), and a combination of both. At the biological level, genetic risk for EDs increases at puberty in boys and girls (Culbert et al., 2009, 2021; Franceschini & Fattore, 2021; Klump, 2013; Timko et al., 2019). This increase is thought to be associated, at least partly, with genetic factors that can be modulated (e.g., activated/deactivated) by sex hormones (Klump, 2013). For example, lower levels of estrogens during puberty have been associated with changes in genetic influences  that may contribute to the risk of ED symptoms (Franceschini & Fattore, 2021; Klump et al., 2018). Additionally, the rapid bodily changes occurring during puberty are central in EDs, where higher body mass index (BMI) represents a biopsychosocial risk factor (Marion et al., 2020; Puhl et al., 2020; van Eeden et al., 2021).

In addition to puberty and reproductive development, body image and self‐esteem may modulate the psychological response to relatively rapid physical changes. For example, children who reach puberty more quickly than their peers (e.g., early menarche in girls) may have more difficulty adjusting emotionally to their physical changes (Zimmer‐Gembeck et al., 2021), which, in turn, may influence their risk for EDs. Beyond biological maturity, considering gendered factors that may be at play during puberty is of interest. For example, it would be insightful to understand how body image and self‐esteem relate to gender/sex, and how their relation may influence the onset of ED symptoms in young adolescents. Some evidence exist on this matter. For example, among youth whose gender identity does not match their assigned sex at birth, puberty may be associated with physical changes that accentuate the discrepancy between their body and gender identity, thereby possibly increasing levels of distress and risk for EDs (Romito et al., 2021).

## 4 NEUROBIOLOGY, GENDER, AND SEX

Sex chromosomes and transcriptional regulatory mechanisms are key to sex expression, as they allow the development of typically masculine (XY) or feminine (XX) traits (Gegenhuber & Tollkuhn, 2019; McCarthy et al., 2017; Swaab et al., 2021). Yet, sex chromosomes can present variations that influence gender/sex in concert with a range of sex hormones and genetic variations. For example, intersex individuals with variations in sexual characteristics often have anatomical characteristics that are not typical of XX or XY. This example illustrates how biological sex cannot invariably be dichotomized. Given their influence on health and well‐being, these sexual variations beyond the typical XX or XY phenotypes must be considered (Rosenwohl‐Mack et al., 2020).

Despite the well‐established variations in gender/sex characteristics, neuroscience research has long conceptualized the masculine and feminine brains as separate entities. This dichotomized categorization might be due to the existence of differences between typically feminine and masculine brains. From as early as fetal development, the brain differentially expresses thousands of genes in men and women (Swaab et al., 2021). Evidence also supports differences in brain size between the sexes, with male brains being 9%–25% larger than female brains (Kaczkurkin et al., 2019). These genetic and neuroanatomical sex differences are thought to be associated with later‐life risk for mental health problems (McCarthy et al., 2017). Yet, the dichotomized categorization of the human brain based on sex has been criticized, as there is considerable intragroup variability among typically “masculine” and “feminine” brains that sometimes outweigh intergroup variability (Fisher et al., 2018; Joel et al., 2015; Kaczkurkin et al., 2019).

It has been proposed that a “brain mosaic” may better represent the link between gender/sex and the human brain. This concept is related to the notion that many traits (e.g., specific behaviors, temperament, and personality traits) are more typically observed in one sex than the other. This notion, however, does not rule out the possibility that individuals can exhibit characteristics more typical of the opposite sex. For instance, physical aggression is typically perceived as a more typically masculine trait, although women can be physically aggressive as well (Hyde, 2014). The concept of brain mosaics applies this notion to neurobiology. It suggests that some brain traits, such as variations in cortical thickness and anatomical or functional connectivity, may be more prevalent in one sex compared to the other (Joel, 2021; Joel et al., 2015). Still, the concept does not preclude the existence of potentially significant neurobiological overlap across sexes.

According to the concept of brain mosaics, only a minority of individuals should exclusively present traits more typical of their own sex (Joel, 2021; Joel et al., 2015). Most individuals would exhibit their own unique combination of typically feminine *and* masculine brain characteristics (Joel, 2021; Joel et al., 2015; Zhang et al., 2021). This notion allows for a wide variability that may, at least partly, reflect the complexity of neurodevelopmental processes in humans that are highly influenced by the gendered social environment (Joel, 2021; Joel et al., 2015; Zhang et al., 2021). Although sex chromosomes and hormones play a role in neurodevelopment and sex/gender differences, several other factors possibly further increase interindividual variability in brain tissues, structures, and functions (Joel, 2021; Joel et al., 2015; Zhang et al., 2021). These factors include gender/sex‐related genetic, epigenetic, growth, and environmental factors, as well as their interaction with timing of exposure, which may contribute to the large variability of phenotypes observed in humans (Joel et al., 2015; Joel, 2021).

Overall, the human brain is clearly far too complex to be dichotomized based on sex; rather, it likely falls along a continuum of masculinity/femininity (Joel et al., 2015; Joel, 2021). The concept of brain mosaics may allow a more inclusive approach to gender‐related variability in EDs research given that brain differences have been reported in gender‐diverse populations (Fisher et al., 2018). How these brain differences fit into a brain mosaic is an interesting area for future study, especially as it relates to refining assessment and treatment approaches in EDs that differ among girls/women, boys/men, and gender nonbinary and gender‐diverse people (Calzo et al., 2018; Hartman‐Munick et al., 2021; Nagata et al., 2020; Strother et al., 2012). Notably, sociocultural gender‐related factors and features have not been directly incorporated in the brain mosaics framework.

## 5 GENDER ROLES AND STEREOTYPES

From an evolutionary perspective, certain features related to traditional gender roles may have been favored in one sex or the other to promote the survival of the human species (Lenroot & Giedd, 2010; Weisberg et al., 2011; Archer, 2019). Gender roles are the sociocultural stereotypes that an individual enacts and adopts throughout life. Some of these features may influence risk for EDs (Weisberg et al., 2011). For example, sensitivity to social cues and empathy were perhaps more favored among women due to their traditional role in the community (e.g., caring for children) (Weisberg et al., 2011; Archer, 2019). Exhibiting more traits related to socialization (i.e., typically feminine traits) may predispose some individuals to EDs via, for instance, an increased vulnerability to other people's socio‐evaluative perceptions. This hypothesis is supported by studies in other psychiatric conditions, such as depressive disorders (Archer, 2019; Rubinow & Schmidt, 2019).

During adolescence, when the first symptoms of EDs often manifest, the social pressures to conform to gender roles increase (Weisberg et al., 2011; Priess et al., 2009). These social pressures may differ by gender/sex and influence risk for EDs throughout development. Likewise, previous studies have reported that individuals who strongly endorse stereotypes related to masculine norms may be at increased risk for mental health problems (Wong et al., 2016), although the opposite has also been reported (Arcand et al., 2020). At the same time, individuals who exhibit both typically feminine and masculine traits, and who are able to adapt and use these traits according to circumstances (i.e., “androgynous” individuals), are thought to be less at risk for mental health difficulties due to their greater adaptability (Juster et al., 2016). In EDs, the endorsement of gender norms and stereotypes also influences treatment‐seeking behaviors (Wong et al., 2016). For example, the endorsement of stereotypes related to masculinity is a barrier to EDs treatment, as seeking help for a condition considered to be “typically feminine” could be seen as compromising masculinity (Murray et al., 2017). Another barrier to EDs treatment is the fear of not being taken seriously by health professionals due to gender stereotypes—a fear that has been reported especially among transgender and other gender‐diverse communities (Hartman‐Munick et al., 2021).

## 6 BODY IMAGE, SEX, AND GENDER

Sex and gender also influence risk for EDs through their relation with body image ideals set by society (Murray et al., 2017; American Psychiatric Association, 2013). Indeed, historical and sociocultural ideals of beauty—muscularity in men and thinness in women—have been associated with risk for ED symptoms (Murray et al., 2017; Lavender et al., 2017; Franceschini & Fattore, 2021; Aparicio‐Martinez et al., 2019; Jiotsa et al., 2021). Body image also affects the risk of EDs in people whose gender identity differs from their sex assigned at birth, sometimes through the search for congruence between body and gender identity (Hartman‐Munick et al., 2021; Romito et al., 2021; Uniacke et al., 2021). For example, a transgender man may restrict his energy intake to limit the development of his breast and maintain a more typically masculine body shape (Murray et al., 2017; Romito et al., 2021; Roberts et al., 2021; Nowaskie et al., 2021).

Other gender‐related motivations may promote the development of ED symptoms. For example, concerns regarding an “ideal” weight for gender affirming surgery might be a preoccupation for some transgender people (Hartman‐Munick et al., 2021). Indeed, obesity is a known risk factor for surgical complications and may prevent some people from accessing gender affirming surgery (Shin et al., 2022; Ives et al., 2019; Brownstone et al., 2021). In addition, BMI can in some cases serve as a reminder to transgender people of the anthropometrics of their sex assigned at birth, which can also be distressing.

Further, individuals whose gender identity does not match their sex assigned at birth and who undergo gender affirming therapy report fewer symptoms of ED than those who do not undergo such therapy (Uniacke et al., 2021). One possible explanation for this finding is that gender affirming therapy may influence body image by better aligning one's physical attributes with their gender identity (Romito et al., 2021). Further, members of the lesbian, gay, bisexual, transgender, and queer (LGBTQ+) community may be confronted with both cisgender beauty standards and that are specific to their community's subcultures (Murray et al., 2017). In sum, the findings presented above point to a need for ED research that addresses the influence of gender and sexual orientation to develop a more comprehensive picture of the risk factors for EDs.

## 7 MINORITY STRESS MODEL AND EATING DISORDERS

The minority stress model refers to health disparities related to the lived experiences of people from stigmatized groups, including (but not limited to) sexual and gender minority (SGM) (DuBois et al., 2021; Uniacke et al., 2021; Meyer, 2003; Juster et al., 2019). Although the underlying biopsychosocial mechanisms remain poorly understood, research has shown that SGM people are more likely to experience high levels of stress (e.g., violence, discrimination, trans/homonegativity, and stigma internalizations) than cisgender and heterosexual people (DuBois et al., 2021; Meyer, 2003; Juster et al., 2019). The compounded forms of stigma that SGM people face may put them at increased risk for mental disorders (DuBois et al., 2021; Uniacke et al., 2021; Meyer, 2003). Other factors may reinforce this stigma, as minority stress is often associated with other risk factors for negative health outcomes (e.g., poverty and social isolation) (Howard et al., 2017). Importantly, victimization experiences are a known risk factor for EDs (Zimmer‐Gembeck et al., 2021; Mitchison et al., 2019; Lie et al., 2019), raising the hypothesis that specific stressors associated with minority stress may also be linked to EDs. Supporting this, SGM individuals who report discriminatory experiences show significantly higher prevalence rates of AN than people who do not report such experiences (3.78% vs. 0.82%) (Kamody et al., 2020). Additionally, some SGM people may use suboptimal coping mechanisms, including avoidance, to cope with the adversity and negative emotions they experience due to stigmatization (Hughto et al., 2017; Brustenghi et al., 2019; Perthes et al., 2021). This use of suboptimal coping strategies may further increase their risk for EDs. In contrast, SGM people who come out to their relatives display better mental health outcomes (Juster et al., 2019), suggesting a positive effect of using adaptive coping mechanisms (e.g., acceptance).

Adverse experiences and stress have the potential to create a biological imprint (DuBois et al., 2021; Juster et al., 2011), possibly through the interaction between biological processes and the social environment. Minority stress may represent a specific form of adversity that can influence important biological mechanisms and increase risk for negative health outcomes in SGM populations (Juster et al., 2015; Juster et al., 2013; Flentje et al., 2020). For example, high levels of stress have been associated with changes in inflammatory markers, and changes in immune/inflammatory processes have been associated with psychiatric disorders, including EDs (McCarthy et al., 2017; VanRyzin et al., 2020; Breton et al., 2022). Further, sex differences in inflammatory profiles and in response to adversity have been identified in previous literature (McCarthy et al., 2017; VanRyzin et al., 2020). By contrast, research shows that “coming out” to family and friends is related to lower diurnal concentrations of the stress hormone cortisol as well as less anxiety, depression, and burnout (Juster et al., 2013). In sum, biological imprinting associated with stressful life experiences can offer an important avenue to explore the biological impact of minority stress and associated risk for psychiatric disorders, including EDs.

## 8 DISCUSSION

**FIGURE 2:**
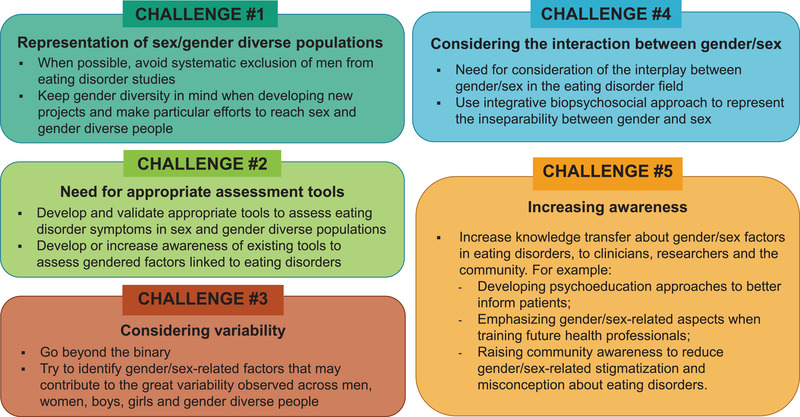
Moving forward with gender/sex research in eating disorders

Overall, there are many ways by which gender and sex can influence risk for EDs. The effect of gender and sex on ED risk fluctuates over the lifetime (Franceschini & Fattore, 2021; Culbert et al., 2021; McCarthy et al., 2017; Rubinow & Schmidt, 2019), which suggests that dynamic and complex interactions between biology and environment are at play. One important current limitation of ED research is that many studies only include presumably cisgender women (Murray et al., 2017; Strother et al., 2012; Coelho et al., 2021). Recruiting cisgender men and gender diverse people in ED research represents a challenge but would be a significant step forward (Coelho et al., 2021). Active multisite collaborative efforts may be needed to optimize recruitment. Still, efforts to integrate gender and sex in ED research should go beyond merely recruiting men and gender‐diverse people (Juster et al., 2016). We propose the following perspectives to help move ED research forward as it relates to gender and sex considerations (Figure 2).

1. *
Representation and participation*. Work is needed to allow the representation of gender/sex‐diverse individuals in ED research and in neuropsychiatry more broadly (Edmiston & Juster, 2022) Large collaborative research efforts might help achieve proper representation. Notably, men are often excluded from research on EDs or, at times, selectively exclude themselves due to gendered expectations. Fortunately, work published in the last 2 years seems to reflect a greater inclusion of gender‐diverse populations in ED research (e.g., males and SGM individuals).
2. *
Measurement and inclusion*. Developing and validating tools to properly characterize gender and sex will help address gender/sex issues in future ED studies. Such work should be done in gender‐diverse populations as well as in community samples, including boys/men, girls/women, and LGBTQ+ people. Given the various biological, developmental, and psychosocial factors associated with gender/sex in EDs, it is imperative that information on sex, gender, sexual orientation, and their correlates be integrated as much as possible into new research. Gender minority individuals should be included in ED research even if subsample sizes are small, as even descriptive statistics are informative and, most importantly, promote inclusion.
3. *
Gender/sex continuums and brain mosaics* . Another approach to move ED research forward would be to conduct studies based on the concept of brain mosaics. In the study of ED symptoms, identifying gender/sex‐related factors (e.g., BMI, temperament, and hormones) that may contribute to the overlap and differences between men, women, and gender diverse people would be enriching. Additionally, going beyond the settled boundaries of “masculinity” and “femininity” based on sex as a binary in neurobiological research might lead to new insights on the neurodevelopmental processes underlying ED symptoms.
4. *
Transdisciplinarity and methodological triangulation* . Integrative approaches incorporating biopsychosocial aspects and considering both gender and sex will promote the advancement of knowledge in biomedical research (Clayton & Tannenbaum, 2016; DuBois et al., 2021; Hankivsky et al., 2018). Developmental studies on EDs including objective measures of puberty, such as hormonal levels (e.g., similar to Lenroot & Giedd (2010) and Juster et al. (2016)), could be useful considering the major role of puberty on ED risk. By addressing sex, gender, and related biopsychosocial aspects in research, it may become possible to clarify their roles in ED risk and treatment response.
5. *
Knowledge transfer and community engagement* . Although the literature is relatively limited, the evidence summarized in the current review suggests that gender/sex affects risk, clinical presentation, and treatment‐seeking behaviors in EDs. Health professionals need to be well informed about the realities of different gender/sex populations.

Knowledge transfer is also key to reduce stigmatization toward communities, such that SGM individuals feel seen and accepted and, most importantly, are not hesitant to seek help for EDs. To ensure that knowledge transfer and health promotion messages have the intended beneficial impact on communities (Tannenbaum et al., 2016), research is needed to design messages that will consider the wide range of gender/sex diversity. Finally, community efforts toward knowledge transfer must be thoughtful and conscientious, and the reinforcement of gender/sex stereotypes in EDs (e.g., EDs are typically feminine conditions) should be avoided (McCarthy et al., 2017).

## 9 CONCLUSION

Sex and gender are increasingly being considered in ED research, and the stereotype that EDs are typically feminine conditions is beginning to dissipate. Nevertheless, current knowledge on EDs in males and LGBTQ+ populations remains quite limited. Further efforts are needed to integrate gender/sex diversity into ED research using a biopsychosocial perspective. We hope that the recommendations proposed in the current review will offer insights and ideas for advancing ED research pertaining to gender/sex considerations and will help in guiding clinical practices and policy‐making for SGM protection.

## CONFLICT OF INTEREST

The authors have no conflict of interest to declare.

### PEER REVIEW

The peer review history for this article is available at https://publons.com/publon/10.1002/brb3.2871.

## Data Availability

Data sharing is not applicable to this article as no new data were created or analyzed in this study.

## References

[brb32871-bib-0001] Allen, K. L. , Byrne, S. M. , La Puma, M. , McLean, N. , & Davis, E. A. (2008). The onset and course of binge eating in 8‐ to 13‐year‐old healthy weight, overweight and obese children. Eating Behaviors, 9(4), 438–446. 18928907 10.1016/j.eatbeh.2008.07.008

[brb32871-bib-0002] American Psychiatric Association . (2013). Diagnostic and statistical manual of mental disorders (5th ed.). American Psychiatric Association.

[brb32871-bib-0003] Aparicio‐Martinez, P. , Perea‐Moreno, A. J. , Martinez‐Jimenez, M. P. , Redel‐Macías, M. D. , Pagliari, C. , & Vaquero‐Abellan, M. (2019). Social media, thin‐ideal, body dissatisfaction and disordered eating attitudes: An exploratory analysis. International Journal of Environmental Research and Public Health, 16(21), 4177. 31671857 10.3390/ijerph16214177 PMC6861923

[brb32871-bib-0004] Arcand, M. , Juster, R. P. , Lupien, S. J. , & Marin, M. F. (2020). Gender roles in relation to symptoms of anxiety and depression among students and workers. Anxiety, Stress and Coping, 33(6), 661–674. 32490683 10.1080/10615806.2020.1774560

[brb32871-bib-0005] Archer, J. (2019). The reality and evolutionary significance of human psychological sex differences. Biological Reviews, 94(4), 1381–1415. 30892813 10.1111/brv.12507

[brb32871-bib-0006] Bankoff, S. M. , & Pantalone, D. W. (2014). Patterns of disordered eating behavior in women by sexual orientation: A review of the literature. Eating Disorder, 22(3), 261–274. 10.1080/10640266.2014.890458 24617312

[brb32871-bib-0007] Breton, E. , Fotso Soh, J. , & Booij, L. (2022). Immunoinflammatory processes: Overlapping mechanisms between obesity and eating disorders? Neuroscience and Biobehavioral Reviews, 138, 104688. 35594735 10.1016/j.neubiorev.2022.104688

[brb32871-bib-0008] Brownstone, L. M. , DeRieux, J. , Kelly, D. A. , Sumlin, L. J. , & Gaudiani, J. L. (2021). Body mass index requirements for gender‐affirming surgeries are not empirically based. Transgender Health, 6(3), 121–124. 34414267 10.1089/trgh.2020.0068 PMC8363993

[brb32871-bib-0009] Brustenghi, F. , Mezzetti, F. A. F. , Di Sarno, C. , Giulietti, C. , Moretti, P. , & Tortorella, A. (2019). Eating disorders: The role of childhood trauma and the emotion dysregulation. Psychiatria Danubina, 31(3), 509–511. 31488781

[brb32871-bib-0010] Calzo, J. P. , Austin, S. B. , & Micali, N. (2018). Sexual orientation disparities in eating disorder symptoms among adolescent boys and girls in the United Kingdom. European Child & Adolescent Psychiatry, 27(11), 1483–1490. 29550905 10.1007/s00787-018-1145-9 PMC6141356

[brb32871-bib-0011] Clayton, J. A. , & Tannenbaum, C. (2016). Reporting sex, gender, or both in clinical research? JAMA, 316(18), 1863–1864. 27802482 10.1001/jama.2016.16405

[brb32871-bib-0012] Coelho, J. S. , Suen, J. , Marshall, S. , Burns, A. , Geller, J. , & Lam, P. Y. (2021). Gender differences in symptom presentation and treatment outcome in children and youths with eating disorders. Journal of Eating Disorders, 9(1), 113. 34526146 10.1186/s40337-021-00468-8 PMC8441244

[brb32871-bib-0013] Cui, H. , Moore, J. , Ashimi, S. S. , Mason, B. L. , Drawbridge, J. N. , Han, S. , Hing, B. , Matthews, A. , McAdams, C. J. , Darbro, B. W. , Pieper, A. A. , Waller, D. A. , Xing, C. , & Lutter, M. (2013). Eating disorder predisposition is associated with ESRRA and HDAC4 mutations. Journal of Clinical Investigation, 123(11), 4706–4713. 24216484 10.1172/JCI71400 PMC3809805

[brb32871-bib-0014] Culbert, K. M. , Burt, S. A. , McGue, M. , Iacono, W. G. , & Klump, K. L. (2009). Puberty and the genetic diathesis of disordered eating attitudes and behaviors. Journal of Abnormal Psychology, 118(4), 788–796. 19899848 10.1037/a0017207 PMC2782672

[brb32871-bib-0015] Culbert, K. M. , Sisk, C. L. , & Klump, K. L. (2021). A narrative review of sex differences in eating disorders: Is there a biological basis? Clinical Therapeutics, 43(1), 95–111. 33375999 10.1016/j.clinthera.2020.12.003 PMC7902379

[brb32871-bib-0016] Dotan, A. , Bachner‐Melman, R. , & Dahlenburg, S. C. (2021). Sexual orientation and disordered eating in women: A meta‐analysis. Eating and Weight Disorders – Studies on Anorexia, Bulimia, and Obesity (EWD), 26(1), 13–25. 10.1007/s40519-019-00824-3 31797331

[brb32871-bib-0017] DuBois, L. Z. , Gibb, J. K. , Juster, R. P. , & Powers, S. I. (2021). Biocultural approaches to transgender and gender diverse experience and health: Integrating biomarkers and advancing gender/sex research. American Journal of Human Biology, 33(1), e23555. 33340194 10.1002/ajhb.23555

[brb32871-bib-0018] Edmiston, E. K. , & Juster, R. P. (2022). Refining research and representation of sexual and gender diversity in neuroscience. Biological Psychiatry: Cognitive Neuroscience and Neuroimaging, 7, 1251–1257. Available from: https://www.sciencedirect.com/science/article/pii/S245190222200177X 35940568 10.1016/j.bpsc.2022.07.007

[brb32871-bib-0019] Favaro, A. , Caregaro, L. , Tenconi, E. , Bosello, R. , & Santonastaso, P. (2009). Time trends in age at onset of anorexia nervosa and bulimia nervosa. Journal of Clinical Psychiatry, 70(12), 1715–1721. 20141711 10.4088/JCP.09m05176blu

[brb32871-bib-0020] Fisher, A. D. , Ristori, J. , Morelli, G. , & Maggi, M. (2018). The molecular mechanisms of sexual orientation and gender identity. Molecular and Cellular Endocrinology, 467, 3–13. 28847741 10.1016/j.mce.2017.08.008

[brb32871-bib-0021] Flentje, A. , Heck, N. C. , Brennan, J. M. , & Meyer, I. H. (2020). The relationship between minority stress and biological outcomes: A systematic review. Journal of Behavioral Medicine, 43(5), 673–694. 31863268 10.1007/s10865-019-00120-6 PMC7430236

[brb32871-bib-0022] Franceschini, A. , & Fattore, L. (2021). Gender‐specific approach in psychiatric diseases: Because sex matters. European Journal of Pharmacology, 896, 173895. 33508283 10.1016/j.ejphar.2021.173895

[brb32871-bib-0023] Gegenhuber, B. , & Tollkuhn, J. (2019). Sex differences in the epigenome: A cause or consequence of sexual differentiation of the brain? Genes, 10(6), 432. 31181654 10.3390/genes10060432 PMC6627918

[brb32871-bib-0024] Grammer, A. C. , Vázquez, M. M. , Fitzsimmons‐Craft, E. E. , Fowler, L. A. , Rackoff, G. N. , Schvey, N. A. , Lipson, S. K. , Newman, M. G. , Eisenberg, D. , Taylor, C. B. , & Wilfley, D. E. (2021). Characterizing eating disorder diagnosis and related outcomes by sexual orientation and gender identity in a national sample of college students. Eating Behaviors, 42, 101528. 34049053 10.1016/j.eatbeh.2021.101528 PMC8380708

[brb32871-bib-0025] Hankivsky, O. , Springer, K. W. , & Hunting, G. (2018). Beyond sex and gender difference in funding and reporting of health research. Research Integrity and Peer Review, 3(1), 6. 30167330 10.1186/s41073-018-0050-6 PMC6112145

[brb32871-bib-0026] Hartman‐Munick, S. M. , Silverstein, S. , Guss, C. E. , Lopez, E. , Calzo, J. P. , & Gordon, A. R. (2021). Eating disorder screening and treatment experiences in transgender and gender diverse young adults. Eating Behaviors, 41, 101517. 33962139 10.1016/j.eatbeh.2021.101517 PMC9645530

[brb32871-bib-0027] Herle, M. , Stavola, B. D. , Hübel, C. , Ferreira, D. L. S. , Abdulkadir, M. , Yilmaz, Z. , Loos, R. J. F. , Bryant‐Waugh, R. , Bulik, C. M. , & Micali, N. (2020). Eating behavior trajectories in the first 10 years of life and their relationship with BMI. International Journal of Obesity, 44(8), 1766–1775. 32461555 10.1038/s41366-020-0581-z PMC7610465

[brb32871-bib-0028] Hildebrandt, T. , Alfano, L. , Tricamo, M. , & Pfaff, D. W. (2010). Conceptualizing the role of estrogens and serotonin in the development and maintenance of bulimia nervosa. Clinical Psychology Review, 30(6), 655–668. 20554102 10.1016/j.cpr.2010.04.011 PMC2910148

[brb32871-bib-0029] Howard, L. M. , Ehrlich, A. M. , Gamlen, F. , & Oram, S. (2017). Gender‐neutral mental health research is sex and gender biased. The Lancet Psychiatry, 4(1), 9–11. 27856394 10.1016/S2215-0366(16)30209-7

[brb32871-bib-0030] Hughto, J. M. W. , Pachankis, J. E. , Willie, T. C. , & Reisner, S. L. (2017). Victimization and depressive symptomology in transgender adults: The mediating role of avoidant coping. Journal of counseling psychology, 64(1), 41–51. 28068130 10.1037/cou0000184 PMC5226079

[brb32871-bib-0031] Hyde, J. S. (2014). Gender similarities and differences. Annual Review of Psychology, 65(1), 373–398. 10.1146/annurev-psych-010213-115057 23808917

[brb32871-bib-0032] Hyde, J. S. , Bigler, R. S. , Joel, D. , Tate, C. C. , & van Anders, S. M. (2019). The future of sex and gender in psychology: Five challenges to the gender binary. American Psychologist, 74(2), 171–193. 30024214 10.1037/amp0000307

[brb32871-bib-0033] Ives, G. C. , Fein, L. A. , Finch, L. , Sluiter, E. C. , Lane, M. , Kuzon, W. M. , & Salgado, C. J. (2019). Evaluation of BMI as a risk factor for complications following gender‐affirming penile inversion vaginoplasty. Plastic and Reconstructive Surgery, 7(3), e2097. 31044103 10.1097/GOX.0000000000002097 PMC6467628

[brb32871-bib-0034] Jiotsa, B. , Naccache, B. , Duval, M. , Rocher, B. , & Grall‐Bronnec, M. (2021). Social media use and body image disorders: Association between frequency of comparing one's own physical appearance to that of people being followed on social media and body dissatisfaction and drive for thinness. International Journal of Environmental Research and Public Health, 18(6), 2880. 33799804 10.3390/ijerph18062880 PMC8001450

[brb32871-bib-0035] Joel, D. (2021). Beyond the binary: Rethinking sex and the brain. Neuroscience and Biobehavioral Reviews, 122, 165–175. 33440198 10.1016/j.neubiorev.2020.11.018

[brb32871-bib-0036] Joel, D. , Berman, Z. , Tavor, I. , Wexler, N. , Gaber, O. , Stein, Y. , Shefi, N. , Pool, J. , Urchs, S. , Margulies, D. S. , Liem, F. , Hänggi, J. , Jäncke, L. , & Assaf, Y. (2015). Sex beyond the genitalia: The human brain mosaic. PNAS, 112(50), 15468–15473. 26621705 10.1073/pnas.1509654112 PMC4687544

[brb32871-bib-0037] Juster, R. P. , Bizik, G. , Picard, M. , Arsenault‐Lapierre, G. , Sindi, S. , Trepanier, L. , Marin, M. F. , Wan, N. , Sekerovic, Z. , Lord, C. , Fiocco, A. J. , Plusquellec, P. , McEwen, B. S. , & Lupien, S. J. (2011). A transdisciplinary perspective of chronic stress in relation to psychopathology throughout life span development. Development and Psychopathology, 23(3), 725–776. 21756430 10.1017/S0954579411000289

[brb32871-bib-0038] Juster, R. P. , de Torre, M. B. , Kerr, P. , Kheloui, S. , Rossi, M. , & Bourdon, O. (2019). Sex differences and gender diversity in stress responses and allostatic load among workers and LGBT people. Current Psychiatry Reports, 21(11), 110. 31630247 10.1007/s11920-019-1104-2

[brb32871-bib-0039] Juster, R. P. , Hatzenbuehler, M. L. , Mendrek, A. , Pfaus, J. G. , Smith, N. G. , Johnson, P. J. , Lefebvre‐Louis, J. P. , Raymond, C. , Marin, M. F. , Sindi, S. , Lupien, S. J. , & Pruessner, J. C. (2015). Sexual orientation modulates endocrine stress reactivity. Biological Psychiatry, 77(7), 668–676. 25444167 10.1016/j.biopsych.2014.08.013 PMC4434405

[brb32871-bib-0040] Juster, R. P. , Pruessner, J. C. , Desrochers, A. B. , Bourdon, O. , Durand, N. , Wan, N. , Tourjman, V. , Kouassi, E. , Lesage, A. , & Lupien, S. J. (2016). Sex and gender roles in relation to mental health and allostatic load. Psychosomatic Medicine, 78(7), 788–804. 27359170 10.1097/PSY.0000000000000351

[brb32871-bib-0041] Juster, R. P. , Smith, N. G. , Ouellet, É. , Sindi, S. , & Lupien, S. J. (2013). Sexual orientation and disclosure in relation to psychiatric symptoms, diurnal cortisol, and allostatic load. Psychosomatic Medicine, 75(2), 103–116. 23362500 10.1097/PSY.0b013e3182826881

[brb32871-bib-0042] Kaczkurkin, A. N. , Raznahan, A. , & Satterthwaite, T. D. (2019). Sex differences in the developing brain: Insights from multimodal neuroimaging. Neuropsychopharmacology, 44(1), 71–85. 29930385 10.1038/s41386-018-0111-z PMC6235840

[brb32871-bib-0043] Kamody, R. C. , Grilo, C. M. , & Udo, T. (2020). Disparities in DSM‐5 defined eating disorders by sexual orientation among U.S. adults. International Journal of Eating Disorders, 53(2), 278–287. 31670848 10.1002/eat.23193

[brb32871-bib-0044] Klump, K. L. (2013). Puberty as a critical risk period for eating disorders: A review of human and animal studies. Hormones and Behavior, 64(2), 399–410. 23998681 10.1016/j.yhbeh.2013.02.019 PMC3761220

[brb32871-bib-0092] Klump, K. L. , Fowler, N. , Mayhall, L. , Sisk, C. L. , Culbert, K. M. , & Burt, S. A. (2018). Estrogen moderates genetic influences on binge eating during puberty: Disruption of normative processes? Journal of Abnormal Psychology, 127(5), 458. 29927265 10.1037/abn0000352 PMC6060616

[brb32871-bib-0045] Lavender, J. M. , Brown, T. A. , & Murray, S. B. (2017). Men, muscles, and eating disorders: An overview of traditional and muscularity‐oriented disordered eating. Current Psychiatry Reports, 19(6), 32. 28470486 10.1007/s11920-017-0787-5 PMC5731454

[brb32871-bib-0046] Lenroot, R. K. , & Giedd, J. N. (2010). Sex differences in the adolescent brain. Brain and Cognition, 72(1), 46. 19913969 10.1016/j.bandc.2009.10.008 PMC2818549

[brb32871-bib-0047] Lie, S. Ø. , Rø, Ø. , & Bang, L. (2019). Is bullying and teasing associated with eating disorders? A systematic review and meta‐analysis. International Journal of Eating Disorders, 52(5), 497–514. 30706957 10.1002/eat.23035

[brb32871-bib-0048] Marion, M. , Lacroix, S. , Caquard, M. , Dreno, L. , Scherdel, P. , Guen, C. G. L. , Caldagues, E. , & Launay, E. (2020). Earlier diagnosis in anorexia nervosa: Better watch growth charts!. Journal of eating disorders, 8, 42. 32905240 10.1186/s40337-020-00321-4 PMC7469097

[brb32871-bib-0049] McCarthy, M. M. , Nugent, B. M. , & Lenz, K. M. (2017). Neuroimmunology and neuroepigenetics in the establishment of sex differences in the brain. Nature Reviews Neuroscience, 18(8), 471–484. 28638119 10.1038/nrn.2017.61 PMC5771241

[brb32871-bib-0050] Meyer, I. H. (2003). Prejudice, social stress, and mental health in lesbian, gay, and bisexual populations: Conceptual issues and research evidence. Psychological Bulletin, 129(5), 674–697. 12956539 10.1037/0033-2909.129.5.674 PMC2072932

[brb32871-bib-0051] Mikhail, M. E. , Anaya, C. , Culbert, K. M. , Sisk, C. L. , Johnson, A. , & Klump, K. L. (2021). Gonadal hormone influences on sex differences in binge eating across development. Current Psychiatry Reports, 23(11), 74. 34613500 10.1007/s11920-021-01287-z PMC8576863

[brb32871-bib-0052] Mitchison, D. , Bussey, K. , Touyz, S. , Gonzalez‐Chica, D. , Musker, M. , Stocks, N. , Licinio, J. , & Hay, P. (2019). Shared associations between histories of victimisation among people with eating disorder symptoms and higher weight. Australian and New Zealand Journal of Psychiatry, 53(6), 540–549. 30501408 10.1177/0004867418814961

[brb32871-bib-0053] Murray, S. B. , Nagata, J. M. , Griffiths, S. , Calzo, J. P. , Brown, T. A. , Mitchison, D. , Blashill, A. J. , & Mond, J. M. (2017). The enigma of male eating disorders: A critical review and synthesis. Clinical Psychology Review, 57, 1–11. 28800416 10.1016/j.cpr.2017.08.001

[brb32871-bib-0054] Nagata, J. M. , Ganson, K. T. , & Austin, S. B. (2020). Emerging trends in eating disorders among sexual and gender minorities. Current Opinion in Psychiatry, 33(6), 562–567. 32858597 10.1097/YCO.0000000000000645 PMC8060208

[brb32871-bib-0055] Nowaskie, D. Z. , Filipowicz, A. T. , Choi, Y. , & Fogel, J. M. (2021). Eating disorder symptomatology in transgender patients: Differences across gender identity and gender affirmation. International Journal of Eating Disorders, 54(8), 1493–1499. 33990998 10.1002/eat.23539

[brb32871-bib-0056] Paolacci, S. , Kiani, A. K. , Manara, E. , Beccari, T. , Ceccarini, M. R. , Stuppia, L. , Chiurazzi, P. , Dalla Ragione, L. , & Bertelli, M. (2020). Genetic contributions to the etiology of anorexia nervosa: New perspectives in molecular diagnosis and treatment. Molecular Genetics & Genomic Medicine, 8(7), e1244. 32368866 10.1002/mgg3.1244 PMC7336737

[brb32871-bib-0057] Perthes, K. , Kirschbaum‐Lesch, I. , Legenbauer, T. , Holtmann, M. , Hammerle, F. , & Kolar, D. R. (2021). Emotion regulation in adolescents with anorexia and bulimia nervosa: Differential use of adaptive and maladaptive strategies compared to healthy adolescents. International Journal of Eating Disorders, 54(12), 2206–2212. 34542185 10.1002/eat.23608

[brb32871-bib-0058] Priess, H. A. , Lindberg, S. M. , & Hyde, J. S. (2009). Adolescent gender‐role identity and mental health: Gender intensification revisited. Child Development, 80(5), 1531–1544. 19765016 10.1111/j.1467-8624.2009.01349.x PMC4244905

[brb32871-bib-0059] Puhl, R. M. , Lessard, L. M. , Larson, N. , Eisenberg, M. E. , & Neumark‐Stzainer, D. (2020). Weight stigma as a predictor of distress and maladaptive eating behaviors during COVID‐19: Longitudinal findings from the EAT study. Annals of Behavioral Medicine, 54(10), 738–746. 32909031 10.1093/abm/kaaa077 PMC7499477

[brb32871-bib-0060] Ramoz, N. , Versini, A. , & Gorwood, P. (2013). Anorexia nervosa and estrogen receptors. Vitamins and Hormones, 92, 141–163. 23601424 10.1016/B978-0-12-410473-0.00006-4

[brb32871-bib-0061] Ridout, S. J. , Ridout, K. K. , Kole, J. , Fitzgerald, K. L. , Donaldson, A. A. , & Alverson, B. (2021). Comparison of eating disorder characteristics and depression comorbidity in adolescent males and females: An observational study. Psychiatry Research, 296, 113650. 33352418 10.1016/j.psychres.2020.113650

[brb32871-bib-0062] Roberts, S. R. , Salk, R. H. , Thoma, B. C. , Romito, M. , Levine, M. D , & Choukas‐Bradley , S. (2021). Disparities in disordered eating between gender minority and cisgender adolescents. International Journal of Eating Disorders, 54(7), 1135–1146. 33638569 10.1002/eat.23494 PMC13051521

[brb32871-bib-0063] Romito, M. , Salk, R. H. , Roberts, S. R. , Thoma, B. C. , Levine, M. D. , & Choukas‐Bradley, S. (2021). Exploring transgender adolescents’ body image concerns and disordered eating: Semi‐structured interviews with nine gender minority youth. Body Image, 37, 50–62. 33549975 10.1016/j.bodyim.2021.01.008 PMC8916039

[brb32871-bib-0064] Rosenwohl‐Mack, A. , Tamar‐Mattis, S. , Baratz, A. B. , Dalke, K. B. , Ittelson, A. , Zieselman, K. , & Flatt, J. D. (2020). A national study on the physical and mental health of intersex adults in the U.S. PLoS One, 15(10), e0240088. 33035248 10.1371/journal.pone.0240088 PMC7546494

[brb32871-bib-0065] Rubinow, D. R. , & Schmidt, P. J. (2019). Sex differences and the neurobiology of affective disorders. Neuropsychopharmacology, 44(1), 111–128. 30061743 10.1038/s41386-018-0148-z PMC6235863

[brb32871-bib-0066] Schorr, M. , & Miller, K. K. (2017). The endocrine manifestations of anorexia nervosa: Mechanisms and management. Nature reviews Endocrinology, 13(3), 174–186. 10.1038/nrendo.2016.175 PMC5998335 27811940

[brb32871-bib-0067] Scott‐Van Zeeland, A. A. , Bloss, C. S. , Tewhey, R. , Bansal, V. , Torkamani, A. , Libiger, O. , Duvvuri, V. , Wineinger, N. , Galvez, L. , Darst, B. F. , Smith, E. N. , Carson, A. , Pham, P. , Phillips, T. , Villarasa, N. , Tisch, R. , Zhang, G. , Levy, S. , Murray, S. , … Schork, N. J. (2014). Evidence for the role of EPHX2 gene variants in anorexia nervosa. Molecular Psychiatry, 19(6), 724–732. 23999524 10.1038/mp.2013.91 PMC3852189

[brb32871-bib-0068] Shin, S. J. , Kumar, A. , & Safer, J. D. (2022). Gender‐affirming surgery: Perioperative medical care. Endocrine Practice, 28(4), 420–424. 35217191 10.1016/j.eprac.2022.02.007

[brb32871-bib-0069] Smink, F. R. E. , van Hoeken, D. , & Hoek, H. W. (2013). Epidemiology, course, and outcome of eating disorders. Eating Disorder, 26(6), 6. 10.1097/YCO.0b013e328365a24f 24060914

[brb32871-bib-0070] Smolak, L. , & Levine, M. P. (2015). Toward an integrated biopsychosocial model of eating disorders. In The Wiley handbook of eating disorders (pp. 929–941). John Wiley & Sons, Ltd.

[brb32871-bib-0071] Stice, E. , Gau, J. M. , Rohde, P. , & Shaw, H. (2017). Risk factors that predict future onset of each DSM‐5 eating disorder: Predictive specificity in high‐risk adolescent females. Journal of Abnormal Psychology, 126(1), 38–51. 27709979 10.1037/abn0000219 PMC5215960

[brb32871-bib-0072] Stice, E. , Marti, C. N. , & Rohde, P. (2013). Prevalence, incidence, impairment, and course of the proposed DSM‐5 eating disorder diagnoses in an 8‐year prospective community study of young women. Journal of Abnormal Psychology, 122(2), 445–457. 23148784 10.1037/a0030679 PMC3980846

[brb32871-bib-0073] Strother, E. , Lemberg, R. , Stanford, S. C. , & Turberville, D. (2012). Eating disorders in men: Underdiagnosed, undertreated, and misunderstood. Eating Disorders, 20(5), 346–355. 22985232 10.1080/10640266.2012.715512 PMC3479631

[brb32871-bib-0074] Swaab, D. F. , & Bao, A. M. (2020). Sex differences in stress‐related disorders: Major depressive disorder, bipolar disorder, and posttraumatic stress disorder. Handbook of Clinical Neurology, 175, 335–358. 33008536 10.1016/B978-0-444-64123-6.00023-0

[brb32871-bib-0075] Swaab, D. F. , Wolff, S. E. C. , & Bao, A. M. (2021). Sexual differentiation of the human hypothalamus: Relationship to gender identity and sexual orientation. Handb Clin Neurol, 181, 427–443. 10.1016/B978-0-12-820683-6.00031-2 34238476

[brb32871-bib-0076] Swanson, S. A. , Crow, S. J. , Le Grange, D. , Swendsen, J. , & Merikangas, K. R. (2011). Prevalence and correlates of eating disorders in adolescents. Archives of General Psychiatry, 68(7), 714–723. 21383252 10.1001/archgenpsychiatry.2011.22 PMC5546800

[brb32871-bib-0077] Tannenbaum, C. , Greaves, L. , & Graham, I. D. (2016). Why sex and gender matter in implementation research. BMC Med Res Methodol, 16(1), 145. 10.1186/s12874-016-0247-7 27788671 PMC5084413

[brb32871-bib-0078] Timko, C. A. , DeFilipp, L. , & Dakanalis, A. (2019). Sex differences in adolescent anorexia and bulimia nervosa: Beyond the signs and symptoms. Current Psychiatry Reports, 21(1), 1. 30637488 10.1007/s11920-019-0988-1 PMC6559358

[brb32871-bib-0079] Treasure, J. , Duarte, T. A. , & Schmidt, U. (2020). Eating disorders. The Lancet, 395(10227), 899–911. 10.1016/S0140-6736(20)30059-3 32171414

[brb32871-bib-0080] Treasure, J. , Stein, D. , & Maguire, S. (2015). Has the time come for a staging model to map the course of eating disorders from high risk to severe enduring illness? An examination of the evidence. Early Intervention in Psychiatry, 9(3), 173–184. 25263388 10.1111/eip.12170

[brb32871-bib-0081] Uniacke, B. , Glasofer, D. , Devlin, M. , Bockting, W. , & Attia, E. (2021). Predictors of eating‐related psychopathology in transgender and gender nonbinary individuals. Eating Behaviors, 42, 101527. 34049054 10.1016/j.eatbeh.2021.101527 PMC8380626

[brb32871-bib-0082] van Eeden, A. E. , Oldehinkel, A. J. , van Hoeken, D. , & Hoek, H. W. (2021). Risk factors in preadolescent boys and girls for the development of eating pathology in young adulthood. International Journal of Eating Disorders, 54(7), 1147–1159. 33682181 10.1002/eat.23496 PMC8359416

[brb32871-bib-0083] VanRyzin, J. W. , Marquardt, A. E. , Pickett, L. A. , & McCarthy, M. M. (2020). Microglia and sexual differentiation of the developing brain: A focus on extrinsic factors. Glia, 68(6), 1100–1113. 31691400 10.1002/glia.23740 PMC8970113

[brb32871-bib-0084] Watson, H. J. , Yilmaz, Z. , Thornton, L. M. , Hübel, C. , Coleman, J. R. I. , Gaspar, H. A. , Bryois, J. , Hinney, A. , Leppä, V. M. , Mattheisen, M. , Medland, S. E. , Ripke, S. , Yao, S. , Giusti‐Rodríguez, P. , Anorexia Nervosa Genetics Initiative , Hanscombe, K. B. , Purves, K. L. , Adan, R. A. H. , … Alfredsson, L. , Eating Disorders Working Group of the Psychiatric Genomics Consortium . (2019). Genome‐wide association study identifies eight risk loci and implicates metabo‐psychiatric origins for anorexia nervosa. Nature Genetics, 51(8), 1207–1214. 31308545 10.1038/s41588-019-0439-2 PMC6779477

[brb32871-bib-0085] Weisberg, Y. J. , Deyoung, C. G. , & Hirsh, J. B. (2011). Gender differences in personality across the ten aspects of the big five. Frontiers in Psychology, 2, 178. 21866227 10.3389/fpsyg.2011.00178 PMC3149680

[brb32871-bib-0086] Wong, Y. J. , Ho, M. H. R. , Wang, S. Y. , & Miller, I. S. K. (2016). Meta‐analyses of the relationship between conformity to masculine norms and mental health‐related outcomes. Journal of counseling psychology, 64(1), 80. 27869454 10.1037/cou0000176

[brb32871-bib-0087] Zeiler, M. , Waldherr, K. , Philipp, J. , Nitsch, M. , Dür, W. , Karwautz, A. , & Wagner, G. (2016). Prevalence of eating disorder risk and associations with health‐related quality of life: Results from a large school‐based population screening. European Eating Disorders Review, 24(1), 9–18. 26010077 10.1002/erv.2368

[brb32871-bib-0088] Zhang, Y. , Luo, Q. , Huang, C. C. , Lo, C. Y. Z. , Langley, C. , Desrivières, S. , Quinlan, E. B. , Banaschewski, T. , Millenet, S. , Bokde, A. L. W. , Flor, H. , Garavan, H. , Gowland, P. , Heinz, A. , Ittermann, B. , Martinot, J. L. , Artiges, E. , Paillère‐Martinot, M. L. , Nees, F. , … Feng, J. (2021). The human brain is best described as being on a female/male continuum: Evidence from a neuroimaging connectivity study. Cerebral Cortex, 31(6), 3021–3033. 33471126 10.1093/cercor/bhaa408 PMC8107794

[brb32871-bib-0089] Zimmer‐Gembeck, M. J. , Webb, H. J. , Kerin, J. , Waters, A. M. , & Farrell, L. J. (2021). Risk factors and temporal patterns of disordered eating differ in adolescent boys and girls: Testing gender‐specific appearance anxiety models. Development and Psychopathology, 33(3), 856–867. 32489165 10.1017/S0954579420000188

